# Simultaneous Hepatic and Mesenteric Hydatid Disease—A Case Report

**DOI:** 10.3389/fsurg.2017.00064

**Published:** 2017-11-21

**Authors:** Daniel Paramythiotis, Anestis Karakatsanis, Petros Bangeas, Konstantinia Kofina, Vassileios Papadopoulos, Stylianos Apostolidis, Antonios Michalopoulos

**Affiliations:** ^1^1st Propedeutic Surgical Department, A.H.E.P.A. University Hospital, Aristotle University Hospital of Thessaloniki, Thessaloniki, Greece

**Keywords:** hydatid cyst, liver diseases, mesentery, drainage cysts, excision surgery, parasitic diseases

## Abstract

**Introduction:**

Hydatid cysts most commonly present in the liver and the lungs; however, they can appear more rarely in other locations, such as the mesentery, with a rather unclear mechanism of manifestation. Herein, we present a case of simultaneous presence of hydatid cysts in the liver and the mesentery of a young man.

**Case report:**

A 39-year-old man was referred to our Department for further investigation of intermittent abdominal pain, especially in the right upper quadrant, and abdominal distension. Abdominal CT imaging revealed three calcified lesions, one in the liver, a similar adjacent to an ileal loop and one close to the urinary bladder, while antibody control was positive for echinococcal infection. The lesions were excised and the patient was discharged on the seventh post-operative day in good general condition. Post-operative control after 6 months did not show any signs of recurrence.

**Conclusion:**

Simultaneous presence of hydatid cysts in two organs occurs in 5–13% of cases. Presence in the mesentery is extremely rare, although, should be included in the classic differential diagnosis, especially in endemic areas.

## Background

Hydatid disease, also referred as echinococcosis, is a chronic, cyst-forming, parasitic helminthic disease.

Three forms of echinococcosis are recognized clinically: cystic echinococcosis caused by *Echinococcus granulosus*, alveolar echinococcosis caused by *Echinococcus multilocularis*, and polycystic echinococcosis caused by *Echinococcus vogeli* or *Echinococcus oligarthrus* (1, 2).

The adult *E. granulosus* resides in the small bowel of the definitive hosts, namely the dogs or other canids. Gravid proglottids release eggs in the feces which, after ingestion by an intermediate host (sheep, goat, swine, cattle, horses, and camel), hatch in the small bowel. These eggs release an oncosphere that penetrates the intestinal wall and migrates through the circulatory system into various organs, especially the liver and lungs (3), but also in more uncommon locations.

In community-based studies, the reported prevalence of cystic echinococcosis ranges from 1 to 7%, while the females and the older population show a greater prevalence than males and other age groups (4).

In most cases, *echinococcosis* is asymptomatic due to the slow growth and development of the cysts. However, the symptoms depending on the size and location of these lesions (5).

Diagnosis of hydatid cyst is achieved by combination of serological tests and imaging, while history of exposure is also important. Imaging findings vary depending on the stage of the cyst (5). In 2001, World Health Organization (WHO) developed a cystic echinococcosis (CE) classification system, in order to deciding the appropriate management worldwide. This system is based on ultrasound imaging findings of cysts. Classical imaging may consist of ultrasound examination, computed tomography (CT), and magnetic resonance imaging (MRI). Calcification of the cysts can be present in 30% of all cases at the time of diagnosis (6).

We describe a case of a male patient presenting simultaneously one hepatic and two intra-abdominal hydatid cysts (one of them in the mesentery), who was treated surgically in our department.

## Case Report

A 39-year-old male patient was referred to our department with mild abdominal distention and pain that was located especially in the right upper abdomen. His symptoms were present during last year, with gradual deterioration. He did not present any fever and occurrence of symptoms was not related to food intake. In his clinical history, he did not mention any previous operations or other medical conditions, whereas he did mention owning two dogs in a rural area.

**Figure 1:**
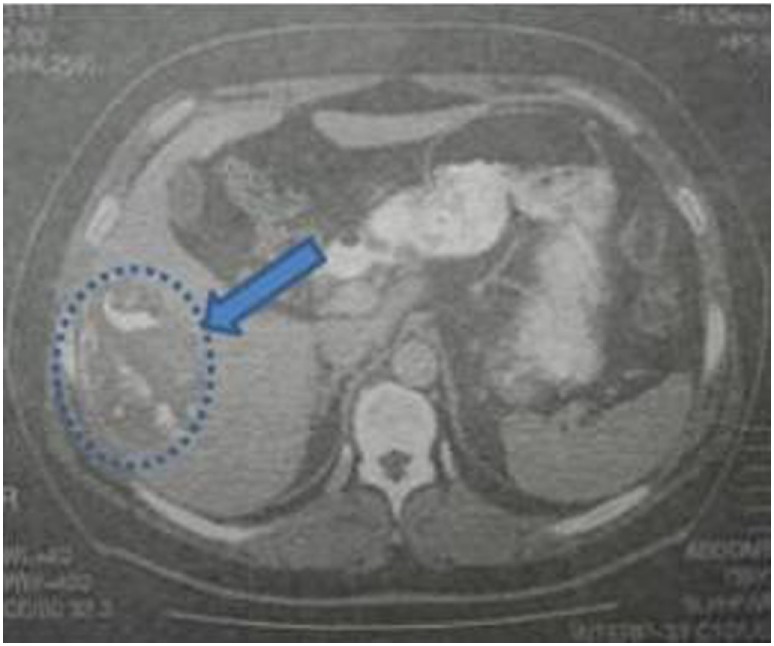
Abdominal computed tomography indicating a hepatic lesion with hyperdense walls, diffuse calcification, and foci of fat in the interior.

**Figure 2:**
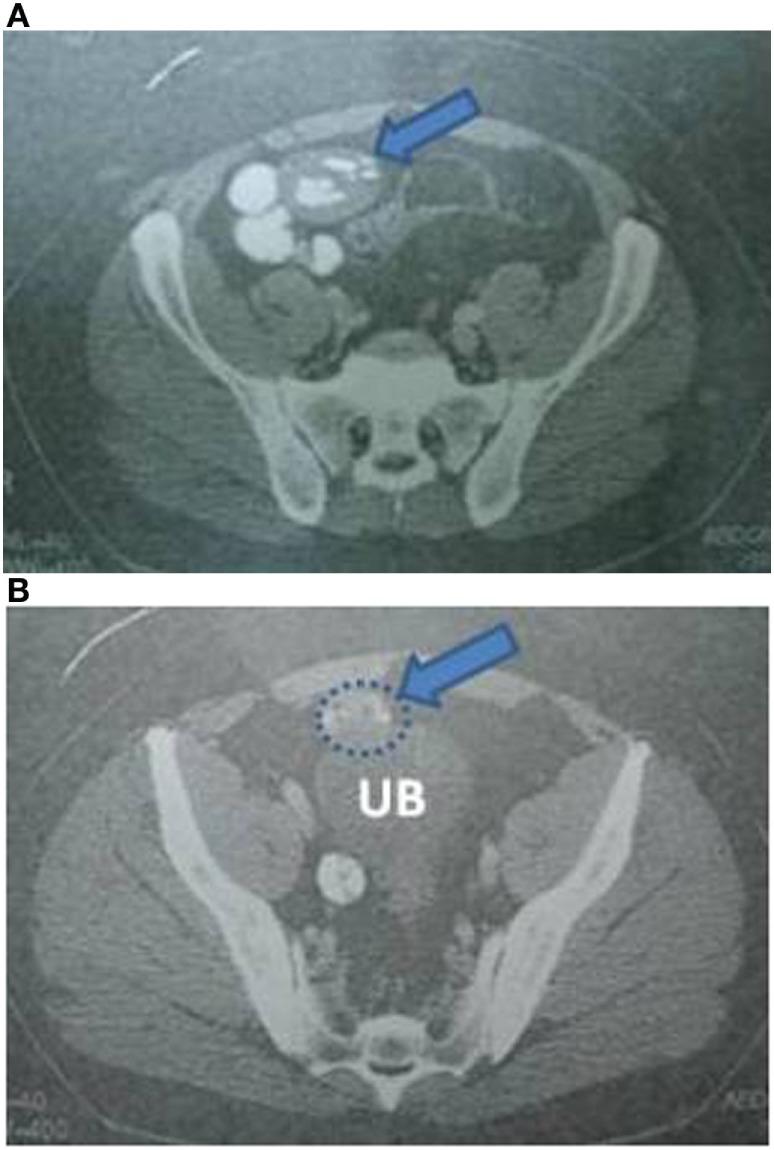
**(A)** Abdominal CT indicating an intra-abdominal cystic lesion, located near an ileal loop. **(B)** Abdominal CT indicating the aforementioned cystic lesion causing external pressure to the urinary bladder, and a smaller cyst at the right side of the wall of the urinary bladder.

**Figure 3:**
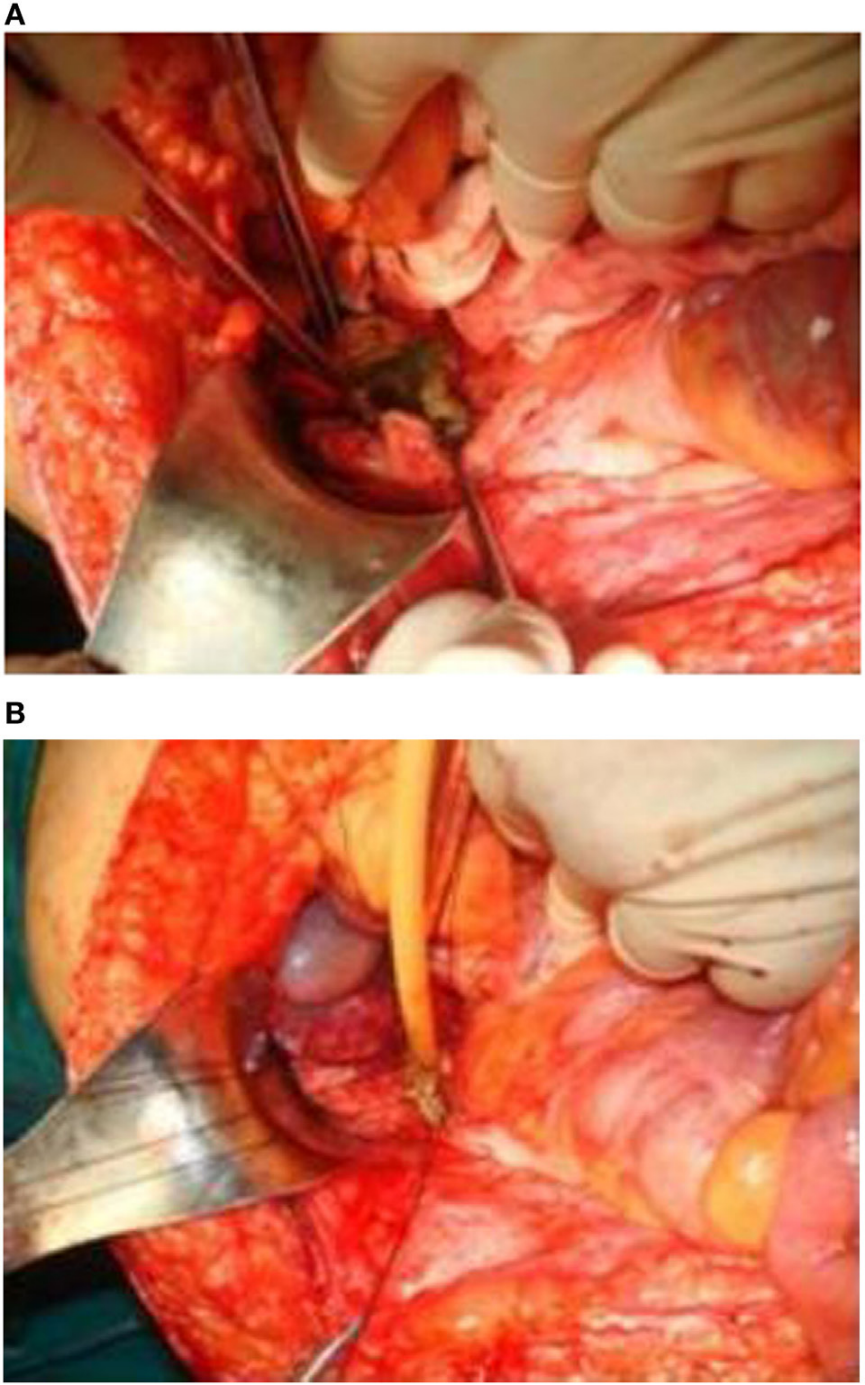
**(A,B)** The hepatic hydatid cyst was opened and drained. A drainage tube was placed in the area.

**Figure 4:**
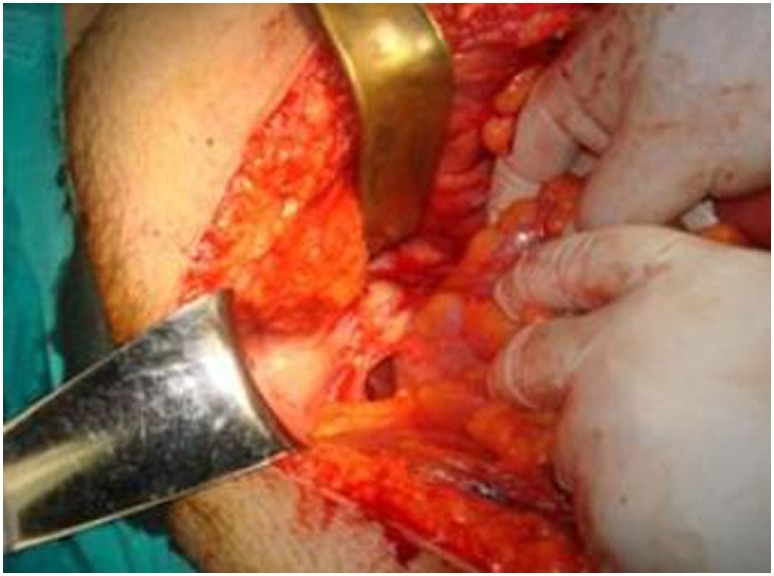
Presence of one hydatid cyst in the mesentery.

**Figure 5:**
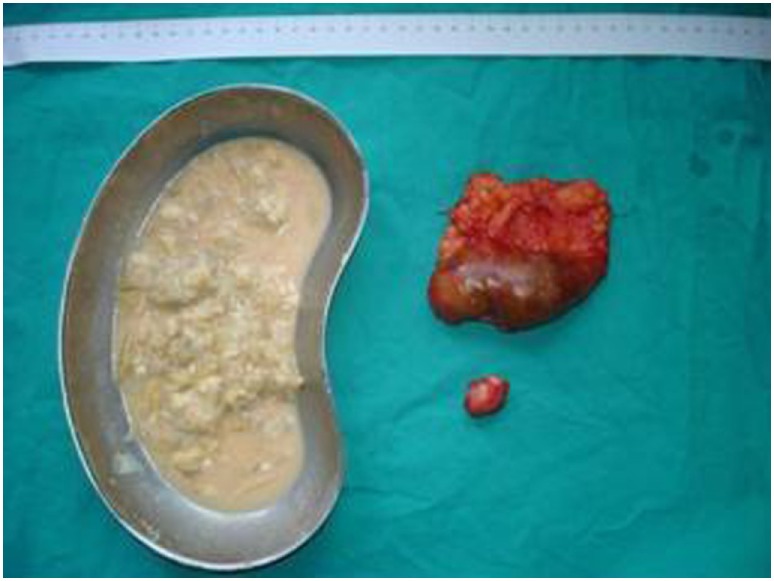
Post-operative specimens. Numerous daughter cysts included in the hepatic cyst near the urinary bladder.

Laboratory blood examination showed results within normal ranges (white blood cells 10.90 K/µL with 57.7% neutrophils, SGOT 20 U/L, SGPT 22 U/L, γ-GT 19 U/L, ALP 111 U/L, and negative quantitative C.R.P. test). Level of eosinophils cells was normal (0.7%), while eosinophilia is present only in 25–40% of all patients. An *Echinococcus* rapid test based on ELISA IgG antibodies detection was positive (quantitative method). Upper abdominal ultrasound revealed a hepatic lesion with vague characteristics. An abdominal CT revealed a large hepatic lesion (9.5 cm × 6.2 cm × 6.8 cm) with a hyperdense wall and multiple internal calcifications (Figure 1). A similar, well circumscribed mass in relation to an ileal loop, that caused external pressure (Figure 2A) to the urinary bladder (5.3 cm × 3.6 cm × 9 cm) and a second, smaller, completely calcified, lesion (2.3 cm × 2 cm × 2.5 cm) near the right side of the urinary bladder (Figure 2B) were also present. Further control for fecal presence of parasites was negative, but blood control for echinococcal antibodies (considering the patient’s history of having dogs in his surroundings and the rather endemic presence of the disease in his area) was positive. All lesions classified according WHO-IWGE (Informal Working Group on Echinococcosis) classification system. After the establishment of the diagnosis, surgical intervention was considered necessary in order to avoid any further abdominal translocation of the parasite. Under general anesthesia, the patient underwent an exploratory laparotomy through midline incision. The hepatic hydatid cyst (CE3a WHO-IWGE) was opened and evacuated from daughter cysts using suction. Washout with hypertonic saline 15% was immediately performed, and a drainage tube was fixed on its location (Figures 3A,B). The other two intra-abdominal lesions, the one found in the mesentery (Figure 4) (CE4 WHO-IWGE) and the smaller near the urinary bladder (CE5 WGO-IWGE), were simply excised (Figure 5). Total length of operation was 125 min. Histopathological findings of mesenteric cyst was possible for primary hydatid cyst, while revealed a lamellated ectocyst and germinal layer with a thick outer, non-cellular membrane in the wall of the cyst. The post-operative period was uneventful and the patient was discharged on the ninth day after the surgery. The patient received albendazole (ABZ) 4 days before surgery (10–15 mg/kg/day) twice a day and was advised to continue treatment with ABZ for 3 months after surgery by 14 days intervals. Pre- and post-operative treatment reduces the risk of recurrence. Three months after surgery, a new chest and abdominal CT was realized, without any signs of recurrence. Patient should be also monitored every 2 weeks with CBC and liver enzyme evaluation for the first 3 months and then every 4 weeks. An *Echinococcus* rapid test based on ELISA sandwich IgG antibodies detection was negative (quantitative method) after 3 months which post-operative treatment with albendazole completed. Follow-up was recommended every 6 months for the first 2 years and then once a year.

## Discussion

Most common sites of hydatid disease are the liver (approximately 70%) and the lungs (15–47%). Kidney (2–4%), bones, and brain are less likely to be involved. Incidence of the disease in other sites, such as the heart, spleen, pancreas, and muscles, is extremely rare. In all cases, involvement of two organs occurs in about 5–13% of the cases (7, 8).

Humans are usually asymptomatic for long period of time while the cysts are growing slowly. In most cases, there is only one cyst, whereas in some cases, multiple cysts may be present (20–40%). Symptoms depend on the size and the number of cysts and possible compression of surrounding structures (9). As in our case, signs of mesenteric hydatid disease may include a non-specific abdominal mass, pain due to traction on the mesentery and pressure effects on adjacent organs.

The signs and symptoms of liver hydatid disease, when present, include hepatomegaly, right upper abdominal or epigastric pain, nausea, and vomiting. Cyst leakage or rupture may lead to systemic immunological responses. Rupture in the peritoneal cavity may also cause secondary disease. In case of involvement with portal vein or biliary tract, cysts may be responsible for segmental or lobar liver atrophy (9). In the case of our patient, the mesenteric hydatid disease was caused possibly by rupture of small daughter cyst from the exterior of the main liver cyst to the peritoneum (10, 11). Although patient had not exhibited signs of anaphylaxis in the past, he had not undergone any other abdominal procedure or suffered from abdominal trauma. Retrograde spread from the liver *via* the hepatic portal vein into the peritoneal cavity was also referred (12).

In the modern era, and with the advent of more sophisticated imaging techniques, such as contrast CT, MRI, and MRCP, there has been a great improvement in the diagnostic accuracy which, in turn, aids even more the clinician to differentiate between other space occupying lesions and to form a treatment plan for the individual patient (13). In addition to imaging, antibody assays are useful to confirm a presumptive radiologic diagnosis (2, 13).

The aim of treatment in hydatid disease is complete elimination of the parasite. Prevention of recurrence and minimization of mortality is essential. Mebendazole (MBZ) and ABZ are the benzimidazole compounds (BMZs) used for the treatment of hydatid disease (2). BMZs may be used alone for the treatment of small (<5 cm) cysts or for inoperable patients. ABZ is considered the drug of choice, because it is more active *in vitro* and it has a better gastrointestinal absorption and bioavailability. The usual dose of orally administered ABZ is 10–15 mg/kg per day in two divided doses; if MBZ is administered, the daily dose is 40–50 mg/kg in three divided doses. The WHO recommends the post-operative administration of ABZ for at least 1 month or MBZ for 3 months but the risk of peritoneal recurrences determines the continuation of the treatment; the longest period reported in the literature was 1 year (5, 14). A perioperative administration of a BMZ agent is also recommended, to reduce the risk of anaphylactoid reactions and prevent recurrence.

Target of surgical procedures is the inactivation of infectious scolices and germinative membranes. Prevention of intra-operative contamination to peritoneal cavity is important while ruptured cysts can lead to systematic anaphylaxis. Recognition and management of complicated cysts requires specialized hepatobiliary surgical team (15). Surgical options may be divided into radical (pericystectomy and organ resection) and conservative approaches (unroofing or capitonnage) (16). In our patient due to the size of the major lesion (liver) and synchronous omentum lesions, we decided to perform abdominal exploration. The liver cyst was opened, sterilized with hypertonic saline washout, and drained with tube placement. The other two intra-abdominal lesions were simply excised. Some randomized trials suggest omentoplasy after cyst removal. In our case, due to involvement of the omentum with peritoneal spread of the disease, we avoided this option (17).

Minimally invasive surgical approaches, utilizing laparoscopic techniques, have been also employed for patients with hydatid disease. There are promising results with laparoscopic treatment but further experience is required (18). Imaging-guided percutaneous puncture, aspiration of the liquid contents, and injection of a protoscolicidal agent (e.g., 95% ethanol or hypertonic saline) for at least 15 min are alternative techniques (2, 19).

Surgery is the treatment of choice of the rupture prone hydatid cysts, all complicated and the most complex cases of liver hydatid disease. Objectives of surgery are (a) inactivating infectious material, (b) preventing contamination, (c) eliminating all viable elements (endocyst), and (d) managing the residual cavity. Surgery is indicated for the treatment of large liver cysts with multiple daughter cysts. As in our case we have a large hepatic cyst and two others in mesentery we choose the open procedure.

Due to the rarity of mesenteric hydatid disease, all the abdominal cystic lesions including mesenteric, pancreatic, gastrointestinal duplication, ovarian cysts, and lymphangiomas must be considered in the differential diagnosis (20).

## Conclusion

Simultaneous presence of cysts on different locations presents a low incidence in hydatid disease, while their presence in the mesentery is rarely reported. In endemic areas, this entity should be included in the differential diagnosis of cysts within the peritoneal cavity. Thorough imaging examination and surgical excision play a key role in the patient’s overall treatment.

## Ethics Statement

Our patient provide his consent for this study with a written informed consent, approved by AHEPA University Hospital of Thessaloniki/Greece Scientific Council.

## Author Contributions

DP, KK, AK, and PB were responsible for the diagnosis and treatment of the patient and assisted in the preparation of the manuscript. VP, SA, and AM provided useful insights.

## Conflict of Interest Statement

The authors declare that the research was conducted in the absence of any commercial or financial relationships that could be construed as a potential conflict of interest.
